# Alcohol and Suicide

**Published:** 1896-11

**Authors:** 


**Alcohol and Suicide.**—At the International Pychological Congress recently held at Munich, a most interesting subject was brought out by Dr. F. C. Mueller, of Munich, in a paper entitled "The Relation of Suicide to Alcohol." He found that both the consumption of alcohol and the number of suicides have increased very much lately. In thickly populated districts, workingmen's towns, the number of suicides is enormous and the alcoholic consumption is positively the cause of the suicide, inasmuch as neurasthenia is caused by alcohol. It is false to believe that beer is not detrimental or less so than whiskey. In countries where whiskey is prohibited, *e. g.*, in Norway, the number of suicides is surprisingly small. The author believed that alcohol so completely demoralizes the human being that suicide is a sort of relief to him. He was sorry to see alcohol introduced into therapeutics, for its ill-effects are far greater than its benefits. He believed alcohol to be a poison, and thought that physicians should take a decided stand against it. In fact, he believed we should be as careful in prescribing alcohol as we are in prescribing morphine or digitalis.—*Berlin Correspondence of N. Y. Medical Record*, October 17, 1896.

